# Satisfaction evaluation of interns with Da Vinci robot surgical demonstration training in gynecologic oncology operations

**DOI:** 10.3389/fpubh.2025.1569153

**Published:** 2025-05-01

**Authors:** Junfen Xu, Yumei Zhou, Mengyan Tu

**Affiliations:** ^1^Department of Gynecologic Oncology, Women’s Hospital, Zhejiang University School of Medicine, Hangzhou, Zhejiang, China; ^2^Laboratory of Innovation and Entrepreneurship, Zhejiang University, Hangzhou, Zhejiang, China

**Keywords:** medical interns, Da Vinci robot surgical demonstration, education evaluation, questionnaires, surgical training

## Abstract

**Objective:**

This study aimed to assess the satisfaction levels of medical interns undergoing training in Da Vinci robot-assisted surgery at the Women’s Hospital.

**Design:**

Between Aug 2023 and Mar 2024, an intern surgical demonstration training was conducted with students in medicine in the Department of Gynecologic Oncology. While the doctor operated from the primary console, a student utilized the 3D perspective from the second console to closely follow the lead surgeon’s surgical maneuvers in real-time. After completing the training, the students filled out a questionnaire to assess their satisfaction with the proposed training.

**Results:**

Through structured questionnaires and interviews, 18 interns’ perceptions and suggestions regarding the training program were evaluated. All students supported the Da Vinci surgical teaching model over traditional teaching methods. The findings revealed the interns’ overall satisfaction with an average five-point Likert scale score of 4.88. Students’ responses highly evaluated the usefulness of Da Vinci surgical training in improving surgical practice capabilities with an average five-point Likert scale score of 4.78. 94% of the students looked forward to using the da Vinci surgical system in future surgeries, and 83% of the participants would continue their further education and training in the field of robotic surgery. Using a 10-point scoring system to evaluate students’ mastery of surgical knowledge gained from this training, the average score obtained was 8.94. Notably, follow-up data revealed that 12 out of these 18 students eventually chose surgery as their specialization when selecting their master’s degree direction.

**Conclusion:**

Training in robot-assisted surgery demonstration of gynecologic oncology operations is a useful approach for training students to master surgical anatomy and develop skills in the field of surgery.

## Introduction

In recent years, the landscape of surgical education has undergone a profound transformation fueled by technological advancements (1, 2). Traditional methods of teaching surgery, reliant on hands-on experience and apprenticeship models, have evolved to incorporate cutting-edge technologies that offer novel avenues for skill development and knowledge acquisition (3–5). Among these innovations, the Da Vinci Surgical System stands out as a groundbreaking platform that revolutionizes the field of surgery (6–9).

Originally designed to facilitate minimally invasive procedures, the Da Vinci Surgical System has garnered widespread acceptance across various surgical specialties, including urology, gynecology, and general surgery (10–12). To date, the fourth generation, the da Vinci Xi Surgical system, was launched in 2014 with the aim of advancing minimally invasive surgery and is widely used in clinical practice (12). Its intuitive interface, ergonomic design, and sophisticated instrumentation enable surgeons to perform intricate procedures with unparalleled precision and controllability. The system’s integration of three-dimensional visualization and robotic assistance further enhances surgical capabilities, providing surgeons with greater dexterity and maneuverability in the operating room. Furthermore, qualitative analysis revealed that trainees advocate the incorporation of robotic training into formal surgical training programs (13). It has also been reported that acquiring surgical skills in surgical trainees and individuals with no prior surgical experience is achieved more rapidly using a robotic platform than a laparoscopic one (14).

The adoption of the Da Vinci Surgical System continues to grow, as does its potential impact on surgical education. Recognizing the transformative potential of this technology, educators are increasingly incorporating Da Vinci demonstrations into surgical training programs. It has been reported that virtual simulator exercises for robot-assisted surgery are valid for assessing innate manual dexterity and can supplement the selection process for surgical training programs (15). Bliznakova et al. reported that training in robotic surgery is beneficial for students aspiring to pursue a career in surgery (16). These demonstrations offer trainees an invaluable hands-on experience and exposure to advanced surgical techniques, preparing them to meet the evolving challenges of modern healthcare.

This study explored the impact of Da Vinci Surgical System demonstrations on surgical education, with a specific focus on skill acquisition, knowledge retention, and overall educational outcomes. We aimed to shed light on the effectiveness of this innovative approach in preparing future surgeons by investigating the satisfaction levels of medical interns undergoing training in Da Vinci robot-assisted surgery demonstrations. Through a comprehensive examination of internal perceptions and feedback, we endeavor to provide insights into the utility and potential areas of improvement for incorporating Da Vinci demonstrations into surgical education curricula.

## Methods

### Participants

The study involved 4^th^-year medical students from Zhejiang University who participated in an intern surgical demonstration training program in the Department of Gynecologic Oncology of Zhejiang University School of Medicine Women’s Hospital from August 2023 to March 2024. Initially, 21 students were randomly selected from the class of 4th-year medical students. However, due to scheduling constraints, 3 students were excluded as their assigned clinical groups did not have exposure to da Vinci surgeries during the observation period. As a result, 18 students participated in the training and completed a satisfaction questionnaire. Although the initial selection process was random, eligibility for inclusion was verified after enrollment, and the questionnaire was administered in a non-anonymous format to assess the impact of the training on students’ choice of specialty for their master’s degree.

### Training program

The intervention surgical demonstration training program was conducted using the da Vinci Xi Surgical System (Intuitive Surgical Inc., Sunnyvale, CA, United States). All participating students had completed a basic surgical training program as part of their standard curriculum, which included exposure to traditional surgical techniques and principles. The training program was structured based on the students’ clinical rotation schedule. It consisted of three sessions conducted over a two-week period. These sessions included 4 h of theoretical background on robotic surgery, 2 h of console practice with the robotic system, and observation of 8–10 live da Vinci surgeries from the second console. The number of surgeries observed per day varied depending on the da Vinci surgery schedule, and the duration of each surgery ranged from 2 to 4 h, based on the complexity and scope of the procedure. The training sessions were led by experienced surgeons specializing in gynecologic oncology. During the training sessions, one student utilized the 3D perspective from the second console to observe and follow the lead surgeon’s maneuvers in real time while the surgeon operated from the primary console. The training focused on mastering the surgical anatomy, providing hands-on experience, and exposure to advanced surgical techniques using the Da Vinci system.

### Questionnaire

After completing the training program, the participants were asked to fill out a structured questionnaire to assess their satisfaction with the training. The questionnaire was conducted after the completion of the clinical rotation and was not related to students’ final assessments or grading outcomes, ensuring impartial responses free from academic pressure. The questionnaire included items designed to evaluate various aspects of the training program, such as usefulness, satisfaction levels, and intention for further education and training in robotic surgery. The questionnaire used a five-point Likert scale to measure participant responses, and a 10-point scoring system to evaluate students’ self-evaluations.

### Data analysis

Responses from the satisfaction questionnaires were collected and analyzed to assess interns’ perceptions and satisfaction levels regarding the training program. Descriptive statistics were used to summarize the data, including the mean scores and percentages of participants indicating satisfaction with different aspects of the training. Additionally, qualitative data from interviews conducted with participants were analyzed to gain deeper insights into their experiences and suggestions for improvement.

### Ethical issues

All participants in the training program provided written informed consent for this study. Ethical approval was obtained from the Zhejiang University School of Medicine Women’s Hospital Ethics Committee (IRB-20240131-R). This study was conducted according to the Declaration of Helsinki.

## Results

**Figure 1:**
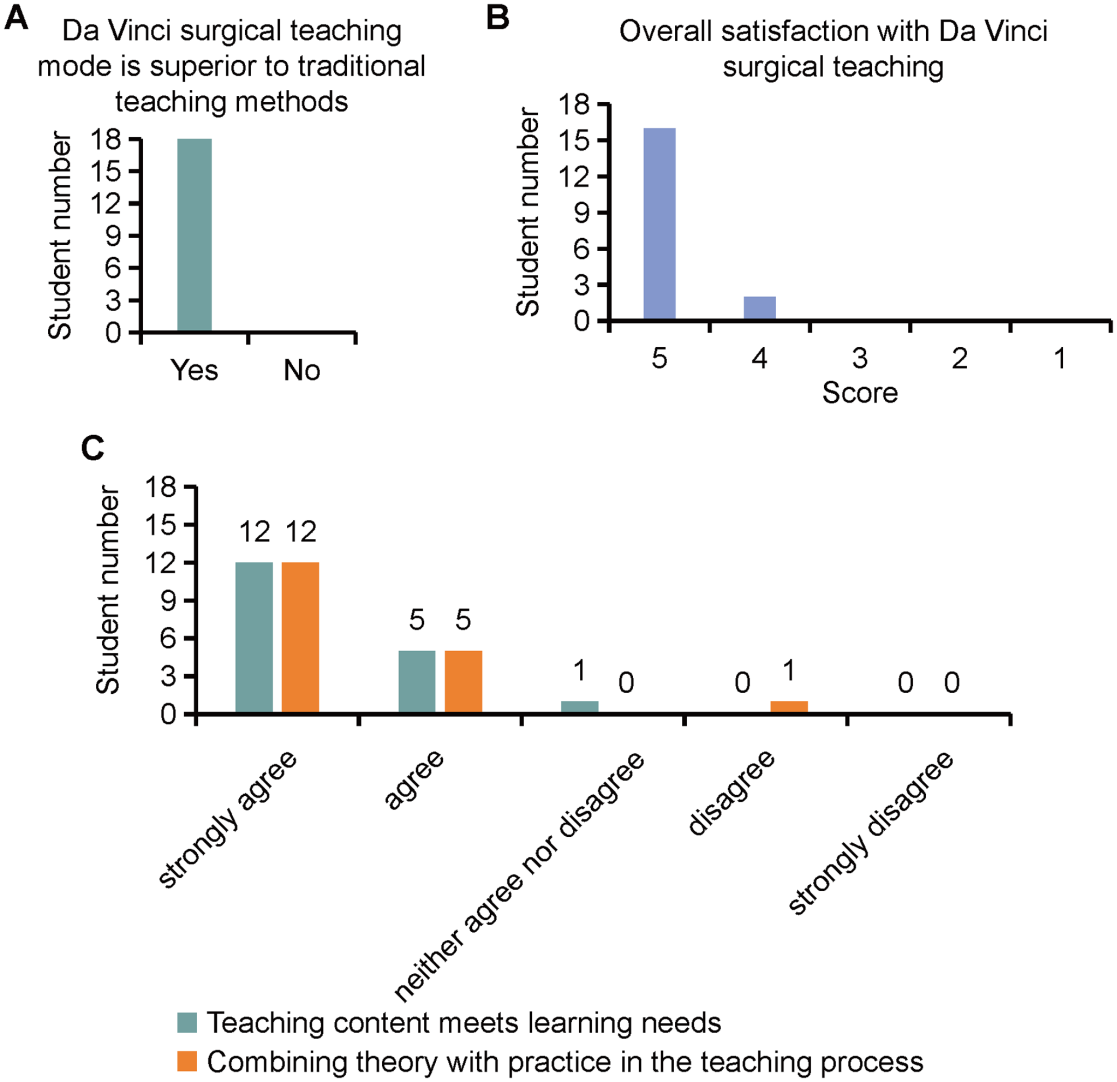
Interns’ satisfaction levels for Da Vinci surgical training program assessed by structured questionnaires.

Eighteen medical interns participated in the Da Vinci robot-assisted surgery demonstration training in the Department of Gynecologic Oncology at the Zhejiang University School of Medicine Women’s Hospital. Following the completion of the training program, interns’ satisfaction levels were assessed using structured questionnaires and interviews. All students rated da Vinci surgical teaching as superior to the traditional teaching methods (Figure 1A). On a five-point Likert scale, the average satisfaction score for the training program was 4.88 (Figure 1B), indicating a high level of overall satisfaction among participants. The results indicated that most students believed that the teaching content met their learning needs and effectively integrated theory with practice during the teaching process (Figure 1C).

**Figure 2:**
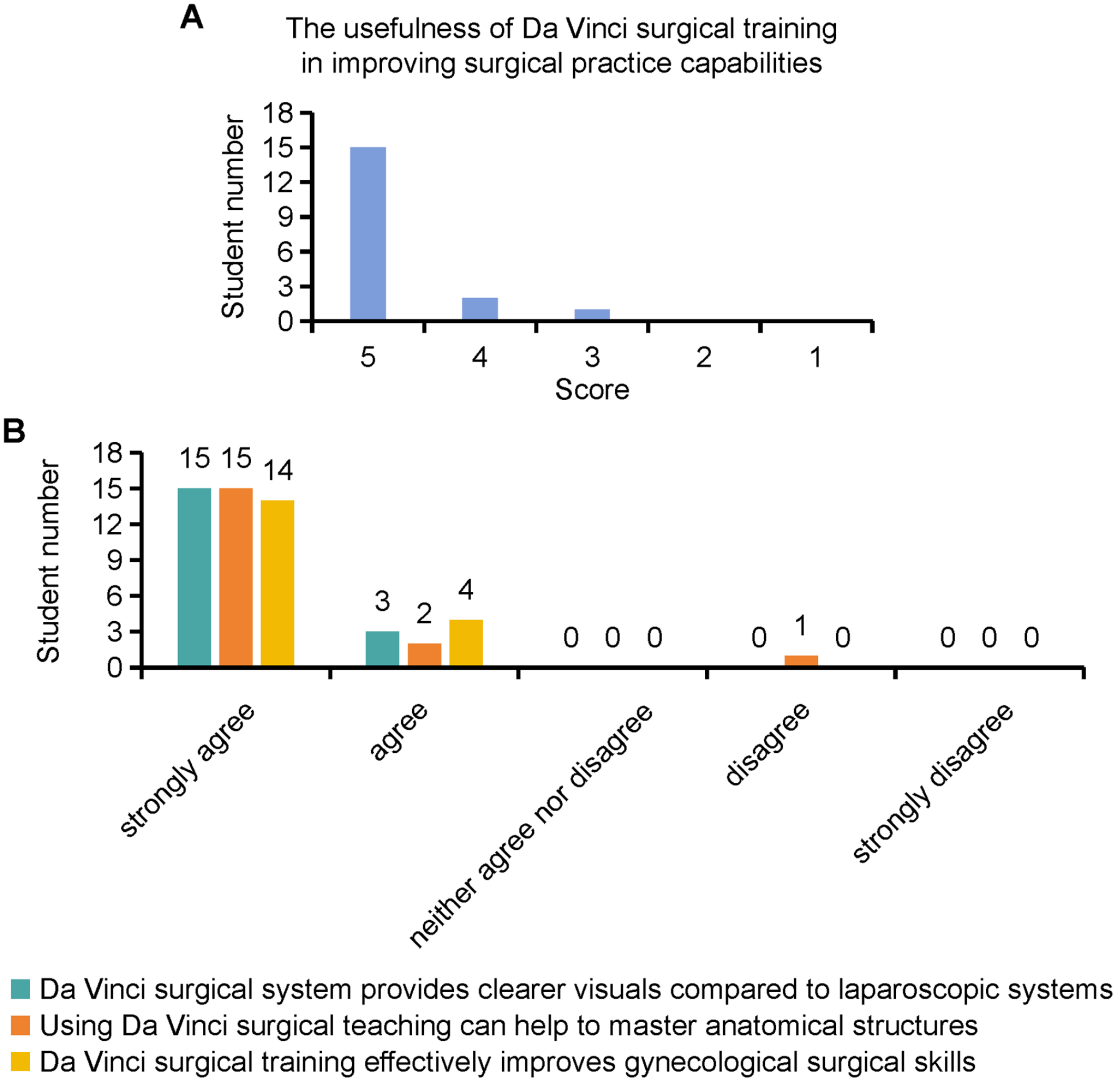
Interns’ evaluation for the usefulness of robotic surgical demonstration training.

The participants were asked to evaluate the usefulness of robotic surgical demonstration training in gynecologic oncology operations (Figure 2A). The average Likert scale score for this aspect was 4.78, suggesting that interns found the training highly beneficial for their skill development and knowledge acquisition. These advantages are specifically manifested in the Da Vinci surgical system, which provides clearer visuals than laparoscopic systems, facilitating the mastery of anatomical structures, and effectively improving gynecological surgical skills during training (Figure 2B). 94% of the students looked forward to using the da Vinci surgical system in their future surgeries. 83% of the students expressed their intention to continue further education and training in the field of robotic surgery. These findings indicate a strong interest among interns in pursuing advanced training opportunities related to robot-assisted surgical techniques.

**Figure 3:**
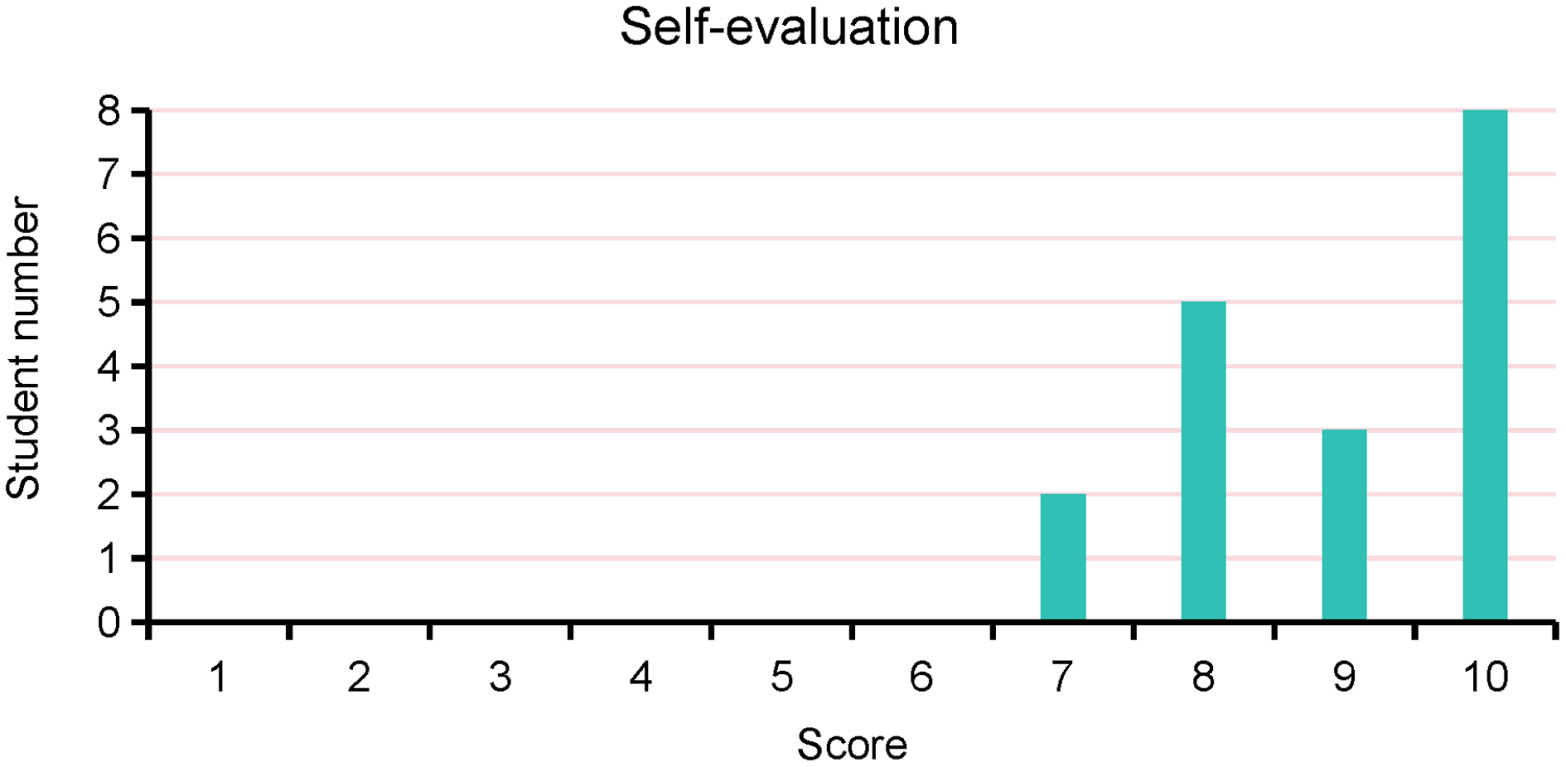
Interns’ self-evaluation for mastery of surgical knowledge gained from this training.

**Figure 4:**
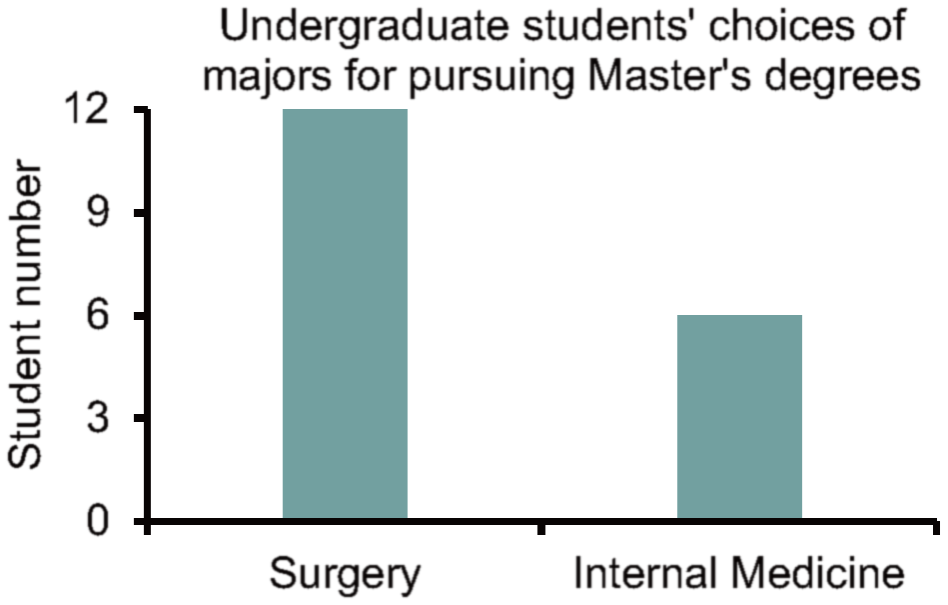
Undergraduate students’ choices of majors for pursuing Master’s degrees.

Through structured questionnaires and interviews, the interns provided insights into their perceptions of the training program and offered suggestions for improvement. Some students believed that Da Vinci surgical teaching was an excellent instructional method, and suggested increasing promotion and innovation efforts. Furthermore, others recommended more frequent utilization of assistant positions because observing from these positions provided better observation and deeper understanding. Common themes included the need for additional hands-on practice sessions, and opportunities for further mentorship and guidance. In addition, the average score obtained from evaluating students’ mastery of surgical knowledge gained from this training, using a 10-point scoring system, was 8.94 (Figure 3). In follow-up assessments, it was found that 12 out of the 18 students ultimately chose surgery as their specialization when selecting their master’s degree direction (67%), while 6 students opted for internal medicine (33%; Figure 4). This outcome highlights the positive influence of the Da Vinci surgical training on students’ career choices and demonstrates the potential of surgical training programs in shaping future medical professionals’ interests and specialties. Taken together, the results suggest that training in robot-assisted surgery demonstrations of gynecologic oncology operations is a valuable approach for preparing medical interns to master surgical anatomy and develop skills in the field of surgery. The high satisfaction levels reported by the participants highlight the effectiveness of incorporating Da Vinci demonstrations into surgical education curricula.

## Discussion

The findings of this study underscore the value of incorporating Da Vinci robot-assisted surgery demonstrations into surgical education, particularly in the context of gynecologic oncology operations. The high satisfaction levels reported by medical interns participating in the training program reflected the effectiveness of this innovative approach in enhancing their learning experience and skill development.

One of the key strengths highlighted by the interns was the usefulness of the robotic surgical demonstration in facilitating their understanding of the surgical anatomy and maneuvers. In a series of well-conducted studies, researchers comprehensively compared 2D, 3D, and 4 K laparoscopic systems in training environments. These studies found that 3D laparoscopy significantly reduced errors in simulated settings for novice surgeons, while 4 K systems provided superior visual comfort with fewer side effects. These findings emphasize the critical role of simulation, checklists, and structured training programs in improving laparoscopic performance and ensuring patient safety (17–19). The immersive nature of the training, with interns closely following the lead surgeon’s actions in real-time from the second console, likely contributed to their ability to grasp complex surgical techniques. Consistent with other reports (20–23), this hands-on exposure to the Da Vinci system’s capabilities not only improved the interns’ knowledge retention, but also instilled confidence in their surgical abilities.

The structured questionnaires and interviews conducted as part of this study provided valuable insights into the interns’ perceptions and suggestions for improving the training program. The overwhelmingly positive responses, with average Likert scale scores exceeding 4 out of 5, indicated a high degree of satisfaction among participants. Furthermore, 94% of the students expressed anticipation of utilizing the Da Vinci surgical system in future surgeries. This favorable reception suggests strong support for the continued integration of Da Vinci demonstrations into surgical education curricula, indicating that such initiatives are well received by students and hold promise for future educational endeavors. Moreover, studies have shown that off-the-shelf instrument tracking systems can validate robotic surgery curricula (24), providing effective assurance for course implementation and feedback. Additionally, the finding that 83% of participants expressed a desire to pursue further education and training in robotic surgery underscores the potential long-term impact of this training program. By inspiring interns to explore career paths in robotic surgery, these demonstrations not only contribute to the development of a skilled workforce, but also drive innovation and advancement in the field of gynecologic oncology. In addition, the follow-up assessments revealed that a significant proportion of the interns chose surgery as their specialization when selecting their master’s degree direction. This distribution suggests that the Da Vinci surgical training program had a notable impact on shaping students’ career paths, particularly in promoting interest in surgical specialties. These findings underline the importance of surgical training programs in fostering students’ professional development and guiding their future career choices, highlighting the potential of such programs to attract and retain talent in the surgical field.

While the results of this study are encouraging, it is essential to acknowledge certain limitations and areas for improvement. For instance, a sample size of 18 participants may limit the generalizability of the findings, warranting further research with larger cohorts to validate the efficacy of Da Vinci demonstrations across diverse student populations. Additionally, future studies could benefit from assessing baseline interest in robotic surgery prior to the training and including a control group for a more robust evaluation of the impact on anatomical knowledge. Furthermore, future iterations of the training program could incorporate objective performance assessments alongside subjective satisfaction measures to provide a more comprehensive evaluation of interns’ skill acquisition and proficiency.

In conclusion, the adoption of Da Vinci robot-assisted surgery demonstrations represents a significant advancement in surgical education, offering trainees a unique opportunity to acquire hands-on experiences with cutting-edge technology. The positive feedback received from the medical interns participating in this study reaffirms the value of such initiatives in preparing future surgeons for the complexities of modern healthcare. Continued investment in innovative educational approaches, coupled with ongoing research and evaluation, will be essential to harness the full potential of robotic surgery in shaping the future of surgical practice and education.

## Data Availability

The data supporting the findings of this study are available upon request from the corresponding author. Requests to access the datasets should be directed to xjfzu@zju.edu.cn.

## References

[1] Brandao LF Autorino R Laydner H Haber GP Ouzaid I De Sio M. Robotic versus laparoscopic adrenalectomy: a systematic review and meta-analysis. Eur Urol. (2014) 65:1154–61. doi: 10.1016/j.eururo.2013.09.021, PMID: 24079955

[2] Ma W Mao Y Zhuo R Dai J Fang C Wang C. Surgical outcomes of a randomized controlled trial compared robotic versus laparoscopic adrenalectomy for pheochromocytoma. Eur J Surg Oncol. (2020) 46:1843–7. doi: 10.1016/j.ejso.2020.04.001 32723609

[3] Custura-Craciun D Cochior D Constantinoiu S Neagu C. Surgical virtual reality - highlights in developing a high performance surgical haptic device. Chirurgia (Bucur). (2013) 108:757–63. 24331310

[4] Hart R Doherty DA Karthigasu K Garry R. The value of virtual reality-simulator training in the development of laparoscopic surgical skills. J Minim Invasive Gynecol. (2006) 13:126–33. doi: 10.1016/j.jmig.2005.11.015, PMID: 16527715

[5] Kyaw BM Saxena N Posadzki P Vseteckova J Nikolaou CK George PP. Virtual reality for health professions education: systematic review and Meta-analysis by the digital health education collaboration. J Med Internet Res. (2019) 21:e12959. doi: 10.2196/12959, PMID: 30668519 PMC6362387

[6] Ismail A Wood M Ind T Gul N Moss E. The development of a robotic gynaecological surgery training curriculum and results of a delphi study. BMC Med Educ. (2020) 20:66. doi: 10.1186/s12909-020-1979-y, PMID: 32131812 PMC7057472

[7] Turner SR Mormando J Park BJ Huang J. Attitudes of robotic surgery educators and learners: challenges, advantages, tips and tricks of teaching and learning robotic surgery. J Robot Surg. (2020) 14:455–61. doi: 10.1007/s11701-019-01013-1, PMID: 31463878 PMC8507581

[8] Hagen ME Inan I Pugin F Morel P. The da Vinci surgical system in digestive surgery. Rev Med Suisse. (2007) 3:1622–6. doi: 10.53738/REVMED.2007.3.117.1622, PMID: 17708229

[9] Sijberden JP Hoogteijling TJ Aghayan D Ratti F Tan EK Morrison-Jones V. International consortium on minimally invasive liver S. Robotic versus laparoscopic liver resection in various settings: an international multicenter propensity score matched study of 10.075 patients. Ann Surg. (2024) 280:108–117. doi: 10.1097/SLA.0000000000006267 38482665 PMC11161239

[10] Adhikari K Penmetsa GK Krishnappa D Taori R Raghunath SK. Revolutionizing urology: the advancements and applications of robotic platforms. J Robot Surg. (2024) 18:106. doi: 10.1007/s11701-023-01758-w, PMID: 38436766

[11] Marengo F Larrain D Babilonti L Spinillo A. Learning experience using the double-console da Vinci surgical system in gynecology: a prospective cohort study in a university hospital. Arch Gynecol Obstet. (2012) 285:441–5. doi: 10.1007/s00404-011-2005-8, PMID: 21779771

[12] Hagen ME Jung MK Ris F Fakhro J Buchs NC Buehler L. Early clinical experience with the da Vinci xi surgical system in general surgery. J Robot Surg. (2017) 11:347–53. doi: 10.1007/s11701-016-0662-0, PMID: 28028750

[13] Fleming CA Ali O Clements JM Hirniak J King M Mohan HM. Surgical trainee experience and opinion of robotic surgery in surgical training and vision for the future: a snapshot study of pan-specialty surgical trainees. J Robot Surg. (2022) 16:1073–82. doi: 10.1007/s11701-021-01344-y, PMID: 34826106 PMC8616984

[14] Gall TMH Alrawashdeh W Soomro N White S Jiao LR. Shortening surgical training through robotics: randomized clinical trial of laparoscopic versus robotic surgical learning curves. BJS Open. (2020) 4:1100–8. doi: 10.1002/bjs5.50353, PMID: 33052038 PMC7709379

[15] Moglia A Morelli L Ferrari V Ferrari M Mosca F Cuschieri A. Distribution of innate psychomotor skills recognized as important for surgical specialization in unconditioned medical undergraduates. Surg Endosc. (2018) 32:4087–95. doi: 10.1007/s00464-018-6146-8, PMID: 29541863

[16] Kalinov T Georgiev T Bliznakova K Zlatarov A Kolev N. Assessment of students' satisfaction with virtual robotic surgery training. Heliyon. (2023) 9:e12839. doi: 10.1016/j.heliyon.2023.e12839, PMID: 36699266 PMC9868440

[17] Restaino S Scutiero G Taliento C Poli A Bernardi G Arcieri M. Three-dimensional vision versus two-dimensional vision on laparoscopic performance of trainee surgeons: a systematic review and meta-analysis. Updat Surg. (2023) 75:455–70. doi: 10.1007/s13304-023-01465-z, PMID: 36811183

[18] Restaino S Paparcura F Arcieri M Pellecchia G Poli A Gallotta V. Employing the aviation model to reduce errors in robotic gynecological surgery: a narrative review. Healthcare (Basel). (2024) 12:1614. doi: 10.3390/healthcare12161614, PMID: 39201172 PMC11353387

[19] Restaino S Vargiu V Rosati A Bruno M Dinoi G Cola E. 4K versus 3D total laparoscopic hysterectomy by resident in training: a prospective randomised trial. Facts Views Vis Obgyn. (2021) 13:221–9. doi: 10.52054/FVVO.13.3.027, PMID: 34555876 PMC8823275

[20] Balasundaram I Aggarwal R Darzi A. Short-phase training on a virtual reality simulator improves technical performance in tele-robotic surgery. Int J Med Robot. (2008) 4:139–45. doi: 10.1002/rcs.181, PMID: 18327876

[21] Lerner MA Ayalew M Peine WJ Sundaram CP. Does training on a virtual reality robotic simulator improve performance on the da Vinci surgical system? J Endourol. (2010) 24:467–72. doi: 10.1089/end.2009.0190, PMID: 20334558

[22] Bric J Connolly M Kastenmeier A Goldblatt M Gould JC. Proficiency training on a virtual reality robotic surgical skills curriculum. Surg Endosc. (2014) 28:3343–8. doi: 10.1007/s00464-014-3624-5 24946742

[23] Valdis M Chu MW Schlachta CM Kiaii B. Validation of a novel virtual reality training curriculum for robotic cardiac surgery: a randomized trial. Innovations (Phila). (2015) 10:383–8. doi: 10.1097/imi.0000000000000222, PMID: 26680752

[24] Tausch TJ Kowalewski TM White LW McDonough PS Brand TC Lendvay TS. Content and construct validation of a robotic surgery curriculum using an electromagnetic instrument tracker. J Urol. (2012) 188:919–23. doi: 10.1016/j.juro.2012.05.005, PMID: 22819403

